# Supplementary material for: Vocal repertoire of Microhyla nilphamariensis from Delhi and comparison with closely related M. ornata populations from the western coast of India and Sri Lanka
Source: PeerJ. 2024 Mar 29;12:e16903. doi: 10.7717/peerj.16903 (PMC10984171; doi:10.7717/peerj.16903)
Supplement: Supplemental Information 5 — Shows eigenvalues for all 20 PC factors, respective proportions of total variance in call properties, cumulative eigenvalues and cumulative variance explained by all factors. [file peerj-12-16903-s005.docx]

| **Factor** | **Eigenvalue** | **% Total variance** | **Cumulative Eigenvalue** | **Cumulative %** |
| --- | --- | --- | --- | --- |
| 1 | 6.060424 | 30.30212 | 6.06042 | 30.3021 |
| 2 | 4.417739 | 22.08869 | 10.47816 | 52.3908 |
| 3 | 3.541808 | 17.70904 | 14.01997 | 70.0999 |
| 4 | 1.808803 | 9.04402 | 15.82877 | 79.1439 |
| 5 | 1.383197 | 6.91599 | 17.21197 | 86.0599 |
| 6 | 0.767745 | 3.83873 | 17.97972 | 89.8986 |
| 7 | 0.711654 | 3.55827 | 18.69137 | 93.4569 |
| 8 | 0.503955 | 2.51978 | 19.19533 | 95.9766 |
| 9 | 0.308202 | 1.54101 | 19.50353 | 97.5176 |
| 10 | 0.237036 | 1.18518 | 19.74056 | 98.7028 |
| 11 | 0.089146 | 0.44573 | 19.82971 | 99.1486 |
| 12 | 0.059799 | 0.29900 | 19.88951 | 99.4475 |
| 13 | 0.045688 | 0.22844 | 19.93520 | 99.6760 |
| 14 | 0.021657 | 0.10829 | 19.95685 | 99.7843 |
| 15 | 0.017802 | 0.08901 | 19.97466 | 99.8733 |
| 16 | 0.010506 | 0.05253 | 19.98516 | 99.9258 |
| 17 | 0.006191 | 0.03096 | 19.99135 | 99.9568 |
| 18 | 0.005250 | 0.02625 | 19.99660 | 99.9830 |
| 19 | 0.001985 | 0.00992 | 19.99859 | 99.9929 |
| 20 | 0.001411 | 0.00705 | 20.00000 | 100.0000 |
