# Supplementary material for: Vocal repertoire of Microhyla nilphamariensis from Delhi and comparison with closely related M. ornata populations from the western coast of India and Sri Lanka
Source: PeerJ. 2024 Mar 29;12:e16903. doi: 10.7717/peerj.16903 (PMC10984171; doi:10.7717/peerj.16903)
Supplement: Supplemental Information 6 — PCA was performed using all 20 measured call properties which generated twenty factors. Shown here are Factor-Call Variable correlations (factor loadings) for all 20 factors. The values in bold with an * define the degree of correlation with corresponding call variable(s). Values for the first five factors are reported in the main text (Table 5). [file peerj-12-16903-s006.docx]

Factor-Call Variable correlations (factor loadings) for factors 1-10.

| Type of Acoustic Property | **Call Property** | Factor 1 | Factor 2 | Factor 3 | Factor 4 | Factor 5 | Factor 6 | Factor 7 | Factor 8 | Factor 9 | Factor 10 |
| --- | --- | --- | --- | --- | --- | --- | --- | --- | --- | --- | --- |
| Call Properties |  |  |  |  |  |  |  |  |  |  |  |
| Temporal call properties | **Call Duration (s)** | **-0.61*** | 0.32 | 0.48 | -0.49 | -0.11 | -0.06 | 0.08 | 0.07 | 0.10 | -0.10 |
|  | **Call Rise Time (s)** | 0.05 | **0.65*** | 0.19 | **-0.66*** | -0.02 | 0.18 | -0.15 | 0.01 | 0.17 | 0.10 |
|  | **Call Fall Time (s)** | **-0.70*** | -0.38 | 0.37 | 0.30 | -0.01 | -0.11 | 0.26 | 0.12 | -0.01 | -0.15 |
|  | **#Pulses per Call** | **-0.74*** | 0.34 | 0.24 | -0.45 | -0.17 | -0.05 | 0.09 | 0.06 | 0.12 | -0.12 |
|  | **Pulse Rate (pulses/s)** | -0.42 | 0.11 | **-0.88*** | -0.04 | -0.09 | 0.06 | 0.03 | -0.02 | 0.01 | 0.01 |
| Spectral call properties | **Overall Dominant Frequency (peak) (kHz)** | 0.11 | **0.68*** | -0.16 | 0.03 | 0.26 | -0.60 | 0.23 | 0.11 | 0.03 | 0.03 |
|  | **Overall Dominant Frequency 1 (peak) (kHz)** | **-0.78*** | 0.36 | 0.05 | 0.40 | 0.07 | 0.08 | -0.21 | -0.08 | 0.11 | 0.10 |
|  | **Overall Dominant Frequency 2 (peak) (kHz)** | **-0.83*** | 0.27 | 0.31 | 0.01 | 0.14 | 0.09 | 0.20 | -0.13 | -0.18 | 0.09 |
| Pulse properties |  |  |  |  |  |  |  |  |  |  |  |
| First, Middle and N-1 Pulses | **First Pulse Period (s)** | 0.19 | -0.01 | 0.30 | 0.14 | **0.76*** | 0.34 | 0.05 | 0.40 | 0.09 | -0.02 |
|  | **Middle Pulse Period (s)** | 0.39 | -0.08 | **0.86*** | -0.09 | -0.01 | -0.10 | -0.22 | -0.03 | -0.14 | 0.01 |
|  | **"N-1" Pulse Period (s)** | 0.41 | -0.16 | **0.63*** | 0.39 | -0.01 | -0.09 | 0.29 | -0.24 | 0.30 | 0.09 |
| Spectral properties maximum pulse | **Overall Pulse Dominant Frequency (kHz)** | 0.23 | **0.85*** | 0.03 | -0.09 | 0.32 | -0.17 | 0.01 | 0.00 | -0.15 | 0.18 |
|  | **Pulse Dominant Frequency 1 (kHz)** | **-0.76*** | 0.42 | 0.12 | 0.36 | 0.05 | 0.09 | -0.18 | -0.13 | 0.11 | 0.08 |
|  | **Pulse Dominant Frequency 2 (kHz)** | **-0.86*** | 0.12 | 0.28 | -0.03 | 0.12 | 0.17 | 0.20 | -0.14 | -0.21 | -0.03 |
| Temporal properties maximum pulse | **Pulse period (s)** | 0.37 | -0.14 | **0.85*** | -0.01 | -0.01 | -0.10 | -0.26 | -0.07 | -0.08 | -0.05 |
|  | **Pulse Duration (ms)** | -0.49 | **-0.71*** | -0.13 | -0.24 | 0.33 | -0.19 | -0.13 | -0.03 | 0.03 | 0.05 |
|  | **Pulse Rise Time (ms)** | 0.48 | 0.30 | -0.29 | -0.16 | 0.56 | 0.06 | 0.00 | -0.41 | 0.04 | -0.26 |
|  | **Pulse 50% Rise Time (ms)** | 0.51 | -0.52 | 0.07 | -0.44 | -0.02 | 0.21 | 0.40 | -0.09 | -0.01 | 0.18 |
|  | **Pulse Fall Time (ms)** | -0.49 | **-0.76*** | -0.09 | -0.23 | 0.24 | -0.19 | -0.13 | 0.00 | 0.03 | 0.04 |
|  | **Pulse 50% Fall Time (ms)** | -0.56 | **-0.73*** | -0.04 | -0.20 | 0.30 | -0.11 | -0.09 | -0.09 | 0.05 | 0.09 |

Supplementary Table 2 (contd.) Factor-Call Variable correlations (factor loadings) for factors 11-20.

| Type of Acoustic Property | **Call Property** | Factor 11 | Factor 12 | Factor 13 | Factor 14 | Factor 15 | Factor 16 | Factor 17 | Factor 18 | Factor 19 | **Factor 20** |
| --- | --- | --- | --- | --- | --- | --- | --- | --- | --- | --- | --- |
| Call Properties |  |  |  |  |  |  |  |  |  |  |  |
| Temporal call properties | **Call Duration (s)** | -0.04 | 0.02 | 0.01 | 0.00 | 0.02 | -0.01 | 0.01 | 0.00 | 0.01 | -0.02 |
|  | **Call Rise Time (s)** | 0.08 | -0.05 | -0.02 | -0.06 | -0.04 | -0.01 | -0.02 | 0.01 | 0.00 | 0.00 |
|  | **Call Fall Time (s)** | -0.10 | -0.02 | -0.02 | -0.07 | -0.04 | -0.01 | -0.02 | 0.01 | 0.00 | 0.00 |
|  | **#Pulses per Call** | -0.04 | 0.06 | 0.01 | 0.05 | 0.01 | 0.02 | 0.00 | -0.01 | -0.01 | 0.02 |
|  | **Pulse Rate (pulses/s)** | -0.03 | 0.03 | -0.10 | 0.01 | 0.01 | -0.01 | 0.01 | 0.04 | -0.01 | 0.00 |
| Spectral call properties | **Overall Dominant Frequency (peak) (kHz)** | 0.04 | -0.11 | 0.00 | 0.01 | 0.02 | 0.00 | 0.01 | 0.00 | -0.01 | 0.00 |
|  | **Overall Dominant Frequency 1 (peak) (kHz)** | -0.07 | -0.04 | 0.02 | 0.04 | 0.03 | -0.03 | -0.04 | -0.01 | -0.01 | 0.00 |
|  | **Overall Dominant Frequency 2 (peak) (kHz)** | 0.06 | 0.06 | -0.02 | -0.06 | 0.05 | -0.02 | 0.02 | -0.02 | -0.01 | 0.00 |
| Pulse properties |  |  |  |  |  |  |  |  |  |  |  |
| First, Middle and N-1 Pulses | **First Pulse Period (s)** | 0.01 | 0.01 | -0.01 | 0.02 | 0.01 | 0.00 | 0.01 | 0.01 | 0.00 | 0.00 |
|  | **Middle Pulse Period (s)** | -0.01 | 0.01 | 0.09 | 0.01 | 0.01 | -0.01 | 0.00 | 0.04 | -0.02 | 0.00 |
|  | **"N-1" Pulse Period (s)** | 0.07 | 0.07 | -0.03 | 0.01 | -0.01 | 0.00 | 0.00 | 0.01 | 0.00 | 0.00 |
| Spectral properties maximum pulse | **Overall Pulse Dominant Frequency (kHz)** | -0.11 | 0.10 | -0.02 | 0.01 | -0.04 | 0.00 | -0.01 | 0.00 | 0.01 | 0.00 |
|  | **Pulse Dominant Frequency 1 (kHz)** | -0.08 | -0.06 | 0.02 | -0.02 | -0.02 | 0.04 | 0.04 | 0.01 | 0.00 | 0.00 |
|  | **Pulse Dominant Frequency 2 (kHz)** | 0.11 | -0.06 | -0.01 | 0.06 | -0.04 | 0.02 | -0.01 | 0.01 | 0.01 | 0.00 |
| Temporal properties maximum pulse | **Pulse period (s)** | -0.04 | -0.05 | -0.15 | 0.01 | 0.02 | 0.01 | 0.00 | -0.01 | 0.00 | 0.00 |
|  | **Pulse Duration (ms)** | 0.00 | 0.03 | 0.01 | -0.03 | 0.03 | 0.06 | -0.03 | 0.01 | 0.00 | -0.01 |
|  | **Pulse Rise Time (ms)** | -0.04 | -0.01 | 0.02 | -0.01 | 0.00 | -0.01 | 0.00 | 0.00 | 0.00 | 0.00 |
|  | **Pulse 50% Rise Time (ms)** | -0.14 | -0.08 | 0.01 | 0.01 | 0.02 | 0.00 | 0.00 | 0.00 | 0.00 | 0.00 |
|  | **Pulse Fall Time (ms)** | 0.00 | 0.01 | -0.02 | 0.03 | -0.06 | -0.02 | 0.02 | -0.02 | -0.02 | 0.00 |
|  | **Pulse 50% Fall Time (ms)** | 0.03 | -0.01 | 0.01 | 0.00 | 0.02 | -0.04 | 0.01 | 0.02 | 0.02 | 0.01 |
