# Supplementary material for: Vocal repertoire of Microhyla nilphamariensis from Delhi and comparison with closely related M. ornata populations from the western coast of India and Sri Lanka
Source: PeerJ. 2024 Mar 29;12:e16903. doi: 10.7717/peerj.16903 (PMC10984171; doi:10.7717/peerj.16903)
Supplement: Supplemental Information 7 — The table shows individuals of both species, Microhyla nilphamariensis and Microhyla ornata being assigned to its respective locality. Microhyla nilphamariensis–Del_01 - Del_18, Microhyla ornata (WC) –WC_01 - WC_05, Microhyla ornata (SL) –SL_01 - SL_05. [file peerj-12-16903-s007.docx]

PC Factor scores for factors 1-10 generated in correlation analyses with call variables. Labelling variable; Locality

| **Case** | Fact. 1 | Fact. 2 | Fact. 3 | Fact. 4 | Fact. 5 | Fact. 6 | Fact. 7 | Fact. 8 | Fact. 9 | Fact.10 | Locality |
| --- | --- | --- | --- | --- | --- | --- | --- | --- | --- | --- | --- |
| **Del_01** | -1.65 | -0.79 | -0.94 | -0.80 | -0.72 | 1.00 | 0.30 | -1.09 | 0.78 | -0.46 | Delhi |
| **Del_02** | -0.65 | 1.56 | -1.47 | -0.84 | -0.97 | 0.85 | -0.38 | 1.20 | -1.05 | -0.50 | Delhi |
| **Del_03** | -0.26 | 0.10 | -0.36 | 1.77 | 0.34 | -0.20 | 2.37 | 0.69 | -0.36 | 1.09 | Delhi |
| **Del_04** | -0.69 | -0.58 | 0.28 | 1.19 | 0.02 | -0.96 | 1.64 | 0.50 | 0.99 | -0.61 | Delhi |
| **Del_05** | 0.52 | 0.25 | 0.75 | 2.86 | -1.09 | 0.27 | -1.14 | -1.21 | -0.57 | -0.76 | Delhi |
| **Del_06** | -0.35 | 1.15 | 1.70 | 0.03 | 1.27 | -0.88 | 0.32 | -0.68 | 0.61 | -1.38 | Delhi |
| **Del_07** | -0.10 | 0.42 | 0.77 | -1.04 | 0.01 | -1.04 | 0.10 | -0.37 | 0.00 | 1.23 | Delhi |
| **Del_08** | 0.43 | 1.43 | 1.28 | -0.05 | -0.32 | 0.14 | -0.20 | 0.87 | 0.19 | -0.77 | Delhi |
| **Del_09** | 0.28 | -0.07 | 2.81 | -0.53 | 0.86 | 2.30 | -0.78 | 0.57 | -1.37 | 1.32 | Delhi |
| **Del_10** | -0.99 | -1.39 | 0.48 | 0.43 | -0.10 | 0.82 | -0.68 | -0.64 | -1.81 | -1.22 | Delhi |
| **Del_11** | -1.04 | -0.58 | -0.11 | -1.27 | -1.39 | 0.99 | 0.51 | -0.92 | 1.33 | 0.48 | Delhi |
| **Del_12** | -0.65 | 1.42 | 0.48 | -0.98 | -0.40 | -1.25 | 2.18 | 0.00 | -2.24 | 0.76 | Delhi |
| **Del_13** | 0.27 | -0.32 | 0.72 | 1.32 | -1.61 | -1.32 | -0.20 | 1.19 | 1.05 | -0.31 | Delhi |
| **Del_14** | 0.21 | 1.74 | 0.76 | -1.38 | -0.09 | -0.37 | -0.39 | -1.52 | 1.94 | -0.67 | Delhi |
| **Del_15** | -1.06 | -0.61 | -0.10 | 0.09 | -0.15 | 1.35 | 0.38 | -0.01 | 0.01 | 0.28 | Delhi |
| **Del_16** | -0.83 | -1.73 | 0.59 | 0.49 | 0.33 | 0.52 | 0.84 | -0.55 | 0.71 | 0.15 | Delhi |
| **Del_17** | -1.76 | -1.47 | 0.03 | -1.22 | 0.26 | -2.23 | -1.83 | 1.88 | -0.57 | -0.90 | Delhi |
| **Del_18** | -0.24 | 0.99 | -0.34 | 0.89 | -1.56 | 0.05 | -1.89 | 0.85 | 0.86 | 1.81 | Delhi |
| **WC_01** | 1.24 | -0.45 | -0.65 | -0.25 | 0.00 | -0.86 | -0.05 | -0.72 | -1.51 | -1.04 | WC |
| **WC_02** | 2.02 | -0.22 | -0.93 | -0.80 | -1.49 | 1.21 | 1.00 | 1.66 | -0.03 | -1.23 | WC |
| **WC_03** | 1.90 | -0.67 | -0.23 | -0.53 | 0.39 | 0.10 | -0.04 | -0.98 | -0.06 | -1.60 | WC |
| **WC_04** | 1.58 | -1.61 | 0.01 | -0.59 | 0.39 | -0.99 | -0.80 | -0.08 | 0.19 | 1.98 | WC |
| **WC_05** | 1.83 | -1.10 | 0.11 | -0.62 | -0.01 | -0.23 | 0.66 | 0.18 | 0.56 | 0.97 | WC |
| **SL_01** | -0.03 | 0.44 | -0.65 | 0.33 | 2.96 | 1.18 | -0.08 | 2.10 | 1.37 | -0.57 | Sri Lanka |
| **SL_02** | 0.18 | 0.93 | -1.00 | 0.46 | 0.04 | 0.27 | -0.43 | -0.47 | -0.38 | 0.33 | Sri Lanka |
| **SL_03** | -0.13 | 0.31 | -1.53 | 0.69 | 1.29 | -0.27 | -0.54 | -0.70 | -0.60 | 0.26 | Sri Lanka |
| **SL_04** | 0.02 | 0.44 | -1.13 | 0.19 | 0.99 | -0.23 | -0.54 | -0.65 | 0.07 | 0.35 | Sri Lanka |
| **SL_05** | -0.06 | 0.42 | -1.31 | 0.18 | 0.74 | -0.21 | -0.34 | -1.09 | -0.10 | 1.00 | Sri Lanka |

Supplementary Table 3 (contd.) PC Factor scores for factors 11-20 generated in correlation analyses with call variables. Labelling variable; Locality

| **Case** | Fact.11 | Fact.12 | Fact.13 | Fact.14 | Fact.15 | Fact.16 | Fact.17 | Fact.18 | Fact.19 | Fact.20 | Locality |
| --- | --- | --- | --- | --- | --- | --- | --- | --- | --- | --- | --- |
| **Del_01** | 0.23 | 2.40 | 0.05 | -0.21 | 0.26 | -0.33 | 0.07 | 0.86 | -1.67 | -0.30 | Delhi |
| **Del_02** | -0.75 | 0.50 | 1.94 | 0.40 | 0.27 | -0.07 | 0.07 | -1.85 | 0.52 | 0.37 | Delhi |
| **Del_03** | -0.14 | 1.19 | 0.26 | 0.99 | -1.60 | 1.17 | -0.79 | -0.35 | -0.97 | 0.88 | Delhi |
| **Del_04** | -1.50 | 0.14 | -0.06 | -0.65 | -0.54 | -0.56 | -0.54 | 0.64 | 1.76 | 0.98 | Delhi |
| **Del_05** | -1.27 | 0.06 | -0.29 | 1.67 | 2.07 | 0.60 | 0.49 | -0.27 | -0.71 | 0.53 | Delhi |
| **Del_06** | -0.14 | 0.24 | 1.69 | 0.43 | 0.12 | 0.80 | 0.33 | 0.19 | 0.88 | -2.57 | Delhi |
| **Del_07** | -0.04 | -0.52 | 3.30 | -0.09 | 0.00 | -0.30 | 0.57 | 0.45 | -0.55 | 1.41 | Delhi |
| **Del_08** | 2.57 | -0.53 | -0.42 | -1.06 | 0.29 | -0.53 | -0.84 | -0.34 | -1.60 | 1.48 | Delhi |
| **Del_09** | -1.39 | 1.16 | -0.70 | -1.01 | -1.06 | 0.28 | -0.01 | -0.03 | 0.02 | -0.36 | Delhi |
| **Del_10** | 0.60 | -0.88 | 0.75 | -0.32 | -0.08 | -0.27 | -1.56 | 0.14 | 0.39 | 0.77 | Delhi |
| **Del_11** | -0.49 | 0.11 | -0.56 | 0.00 | 0.52 | 0.17 | 0.56 | -1.61 | -0.19 | -0.77 | Delhi |
| **Del_12** | -0.02 | -0.57 | -1.89 | 0.73 | 1.14 | -1.48 | 0.47 | 0.21 | -0.11 | -0.83 | Delhi |
| **Del_13** | 0.54 | -0.01 | -0.06 | -1.48 | -0.67 | -0.19 | 0.18 | -1.34 | -1.34 | -1.75 | Delhi |
| **Del_14** | -0.56 | -0.02 | -1.22 | 0.42 | -0.12 | 0.79 | -1.29 | 0.93 | 0.25 | 1.35 | Delhi |
| **Del_15** | 2.20 | -1.82 | -0.03 | 2.20 | -0.84 | 1.29 | 0.03 | 0.86 | 0.34 | -0.91 | Delhi |
| **Del_16** | 0.34 | -1.04 | 0.13 | -1.43 | 1.01 | -1.43 | 0.75 | -0.12 | 1.13 | 0.58 | Delhi |
| **Del_17** | -0.88 | -0.20 | -1.09 | 0.60 | -0.35 | 1.11 | 0.48 | 0.20 | -0.39 | 0.65 | Delhi |
| **Del_18** | 0.62 | 0.76 | -0.27 | 0.79 | -0.89 | -1.67 | 0.12 | 1.32 | 1.96 | -0.11 | Delhi |
| **WG_01** | 1.92 | 2.51 | -0.25 | -0.66 | 0.50 | 0.44 | 0.06 | -0.12 | 1.96 | -0.10 | WG |
| **WG_02** | -1.30 | -1.10 | 0.22 | -0.56 | 0.59 | 0.46 | -1.11 | 1.73 | 0.13 | -0.74 | WG |
| **WG_03** | -0.45 | -0.41 | -0.23 | 1.52 | -2.65 | -2.28 | 1.29 | -0.89 | -0.56 | 0.26 | WG |
| **WG_04** | -0.11 | -0.30 | 0.55 | 0.83 | 1.39 | -0.55 | -1.26 | -0.13 | -0.47 | -1.09 | WG |
| **WG_05** | 0.65 | 0.59 | -0.19 | 0.27 | 0.37 | 1.81 | 1.18 | -0.05 | 0.10 | 1.17 | WG |
| **SL_01** | 0.36 | 0.40 | -0.40 | 0.78 | 1.62 | -0.80 | 0.60 | -0.53 | 0.02 | 0.13 | Sri Lanka |
| **SL_02** | -0.06 | -1.37 | -0.39 | -1.73 | -0.43 | 1.47 | 2.92 | 0.48 | 0.29 | 0.05 | Sri Lanka |
| **SL_03** | -0.39 | 0.23 | 0.23 | -0.97 | -0.04 | -0.75 | 0.10 | 2.36 | -1.89 | -0.53 | Sri Lanka |
| **SL_04** | 0.05 | -0.54 | -0.43 | -0.77 | -1.12 | 0.71 | -1.83 | -0.92 | 0.27 | -1.25 | Sri Lanka |
| **SL_05** | -0.57 | -0.97 | -0.65 | -0.66 | 0.23 | 0.12 | -1.05 | -1.81 | 0.41 | 0.70 | Sri Lanka |
